# Bending resistance and cyclic fatigue life of Reciproc Blue, WaveOne Gold, and Genius files in a double (S-shaped) curved canal

**DOI:** 10.15171/joddd.2017.042

**Published:** 2017-12-13

**Authors:** Taha Özyürek, Mustafa Gündoğar, Koray Yılmaz, Gülşah Uslu

**Affiliations:** ^1^Department of Endodontics, Faculty of Dentistry, Ondokuz Mayis University, Samsun, Turkey; ^2^Department of Endodontics, Faculty of Dentistry, Medipol University, Samsun, Turkey; ^3^Samsun Oral and Dental Health Hospital, Samsun, Turkey

**Keywords:** Cyclic Fatigue, Cross-section Area, Double Curvature, Genius File, Reciproc Blue, WaveOne Gold

## Abstract

***Background.*** This study compared the cyclic fatigue resistance, bending resistance and cross-sectional areas of Reciproc Blue (RPC Blue), WaveOne Gold (WOG), and Genius File (GF) NiTi rotary systems.

***Methods.*** Forty RPC Blue R25 (25/.08), 40 WOG Primary (25/.07) and 40 GF (25/.04) files were used in the present study. Flexibility of the files was determined by 45° bending test. The instruments were also subjected to cyclic fatigue resistance, calculating the number of cycles to fracture (NCF) in an S-shaped artificial stainless steel canal. Also the cross-sectional areas of the files were measured at D5 level. The length of the fractured file tips was measured. The fracture surface of all the fragments was examined with a scanning electron microscope. Data was statistically analyzed using one-way ANOVA and post hoc Tukey tests.

***Results.*** In both the apical and coronal curvatures, the NCF of the GF was significantly higher than that of the RPC Blue and WOG files. There was no significant difference between the GF, WOG and Reciproc Blue files with respect to the lengths of the fractured file fragments in either the apical or coronal curvature. The bending resistance of the GF was signif-icantly higher than that of the RPC Blue and WOG files. The RPC Blue had the largest cross-sectional area, and the GF had the smallest cross-sectional area.

***Conclusion.*** Within the limitations of the present study, the GF NiTi system exhibited the highest cyclic and bending re-sistance among the experimental groups.

## Introduction

Nickel-titanium (NiTi) file fracture during root canal preparation procedures is one of the most frequently seen complications.^1,2^ Removing the fractured segment of the file, which is stuck into the root canal, is generally a difficult process, and the amount of residual dentin tissue significantly decreases during this procedure. As a result, the prognosis of endodontic treatment might be negatively affected.^3^ Currently, considering the many advantages of NiTi files, the use of NiTi rotary file systems has become popular for shaping the root canals. However, despite those numerous advantages, the NiTi rotary files might fracture, especially within the curved root canals, due to cyclic fatigue.^2^ The manufacturers make efforts to improve the fracture resistance of NiTi files by altering the designs, applying various heat treatments, and using various alloys.^4^ In addition, changing the kinematics of NiTi rotary files may also alter fracture resistance of the files. It was shown that the stress on files, which performs preparation via reciprocation motion, is less than the stress on those with continuous rotation motion and thus NiTi file’s cyclic fatigue resistance increases.^5^

Reciproc Blue (RPC Blue; VDW, Munich, Germany) and WaveOne Gold (WOG; Dentsply Maillefer, Baillagues, Switzerland) are a new generation single-file systems that have recently been introduced to the market. Both file systems have reciprocating motion. RPC Blue is the latest version of Reciproc (RPC; VDW) files. In common with RPC, RPC Blue files have an S-shaped cross-section, two cutting edges and a non-cutting tip. A novel aspect of the new RPC Blue file system is the molecular structure of the files. This has been altered using a new type of heat treatment, which increases the cyclic fatigue resistance of the files and gives the files a blue color. According to the manufacturer, the cyclic fatigue resistance of RPC Blue files is approximately twice that of RPC files.^6^

WOG files are the most recent version of the earlier WO (WO; Dentsply Maillefer) files. WOG files retain the reciprocating motion of WO files but feature different dimensions, cross-sections and geometries. The cross-section of the WOG file has also been modified, so that it is now a parallelogram, with two cutting edges. In addition, the off-center design used in ProTaper Next (Dentsply Maillefer) files has been incorporated in WOG files. WOG files are manufactured using gold heat treatment technology. In files produced using M-Wire technology, the heat treatment step is carried out before manufacturing the files. In contrast, in gold heat treatment, the files are heated and then slowly cooled after they have been manufactured. The manufacturer claims that the new heat treatment increases the flexibility of the files.^7^

Genius File (GF; Ultradent, South Jordan, UT, USA) is a file system, which was recently introduced to the market. It is made of heat-treated NiTi alloy and operates based on both reciprocation and rotation motions. The manufacturer recommends the use of the file with a 0.25-mm apical diameter in reciprocation motion and others (with 0.30, 0.35, 0.40, and 0.50 mm diameters at D0 point) in reciprocation motion up to the working length and then in rotational motion.^8^ The reciprocation motion of GF differs from those of RPC Blue and WOG files. In contrast, GF performs its reciprocation motion first in clockwise and then in counterclockwise directions, and GF reciprocation angle (90° clockwise and 30° counterclockwise) is less that that with RCP Blue and WOG.

There are no studies in the literature on the bending and cyclic fatigue resistance of the novel Genius File (GF) NiTi system. The aim of the present study was to compare the cyclic fatigue resistance, bending resistance and cross-sectional areas of WOG, RPC Blue, and GF systems, with different angles of reciprocation. The null hypotheses tested were as follows:

1. There would be no difference in the bending resistance of the instruments.
2. There would be no difference in the cyclic fatigue resistance of the instruments.

## Methods

A total of 120 NiTi rotary files were included in the present study. Twenty RPC Blue R25 (25/.08) (lot no: 176554), 20 WOG Primary (25/.07) (lot no: 1323197) and 20 GF (25/.04) (lot no: 017117) files were used in the static cyclic fatigue test. Twenty RPC Blue R25 (25/.08) (lot no: 176554), 20 WOG Primary (25/.07) (lot no: 1323197) and 20 GF (25/.04) (lot no: 017117) were used in the bending resistance test and cross-sectional area analysis. For standardization and reliability of the experiment, defects or deformities in the tested instruments were assessed under a stereomicroscope (Olympus BX43; Olympus Co, Tokyo, Japan) before the experiment. No irregularities were found in any of the instruments. Thus, all the instruments were included in the study.

### Cyclic fatigue test

The instruments were tested within an artificial canal, with a double curvature (coronal and apical curves). The coronal curve had a 60° angle relative to the curvature and a radius of curvature of 5 mm. The curvature was located 8 mm from the tip of the canal. The apical curve had a 70° angle relative to the curvature and a radius of curvature of 2 mm, the center of which was located 2 mm from the tip of the canal.^9^ In all the groups, the files were lubricated with a synthetic lubricant (WD-40 Company, Milton Keynes, U.K.) to minimize friction between the canal walls and the files to ensure free rotation of the files within the artificial canal. The upper part of the artificial canal was open, and it was covered with tempered glass to prevent the instruments from slipping out. The files were divided into the following three groups for the cyclic fatigue test.

### Group 1: RPC Blue R25

The files were operated using a VDW Reciproc Gold (VDW) endodontic motor, which was mounted on a cyclic fatigue test device. The “Reciproc ALL” program was used until fracture occurred.

### Group 2: WOG Primary

The files were operated using a VDW Reciproc Gold (VDW) endodontic motor, which was mounted on a cyclic fatigue test device. The “WaveOne ALL” program was used until fracture occurred.

### Group 3: GF 25/.04

The files were operated using EndoEZE Genius (Ultradent) endodontic motor, which was mounted on a cyclic fatigue test device. The “Reciprocation” program (90° clockwise and 30° counter clockwise movements at 350 rpm) was used until fracture occurred.

The number of cycles to fracture (NCF) for each file was calculated using the formula: NFC = rotation speed (rpm) × time (sec)/60. The length of the fractured file tips in the apical and coronal thirds was measured using a digital micro caliper.

**Figure 1:**
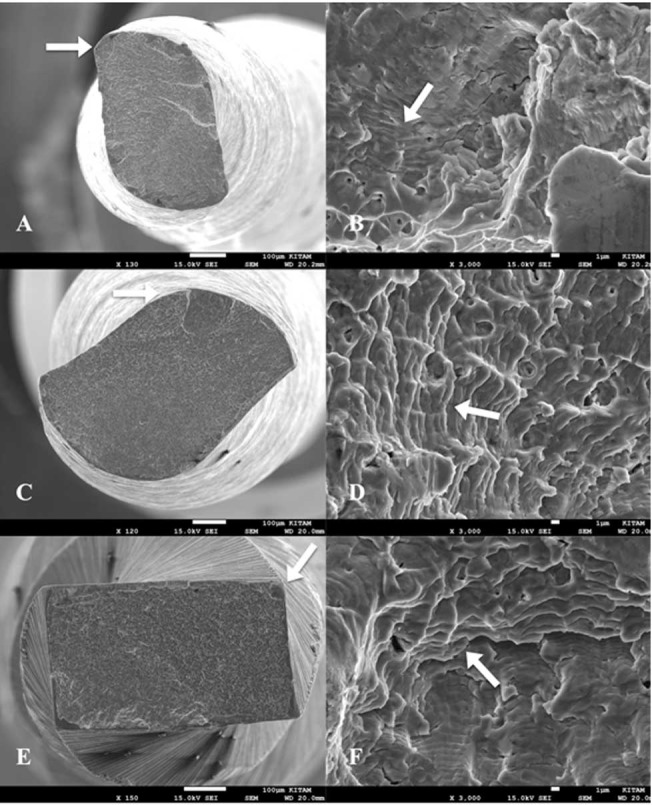
Scanning electron microscopic appearances of Genius, Reciproc Blue and WaveOne Gold files after cyclic fatigue testing. Fractured surface view of (A) Genius File, (C) Reciproc Blue and (E) WaveOne Gold and high-magnification view of (B) Genius File, (D) Reciproc Blue and (F) WaveOne Gold instruments showing fatigue striations typical of cyclic fatigue (white arrows).

In total, six pieces of fractured files (two pieces from each group) were examined under a scanning electron microscope (SEM; JEOL, JSM-7001F, Tokyo, Japan) to determine the fracture types of the files. Photomicrographs of the fractured surfaces were taken under different magnifications (Figure 1).

### Bending resistance test

The bending resistance of the 20 instruments in each group (RPC Blue, WOG, and GF) was investigated using an Instron universal testing machine (Instron, Canton, MA). A 20-N load cell was attached to the machine. The test was performed at 15 mm/min, using a flexible stainless steel wire.^10^ One end of the wires was fastened to the testing machine head, and the other end was attached 3 mm from the instrument tip, as described previously.^10^ Using a cable, the instruments were fixed at an angle of 45° relative to the machine base, so that their tips were tensioned until they reached the plane parallel to the base, coincident with the fixing point. The moment of bending at an angular deflection of 45° was recorded.

### Determination of the cross-sectional areas of the instruments

**Figure 2:**
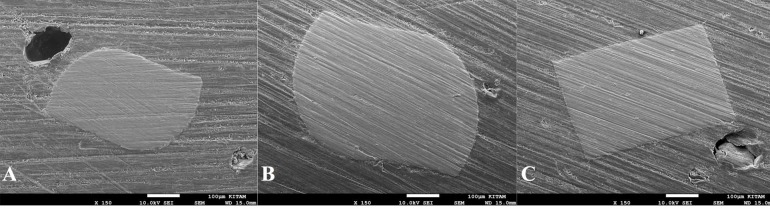
Cross-sectional SEM appearances of (A) Genius File, (B) Reciproc Blue and (C) WaveOne Gold at D5 level.

The instruments were embedded vertically in an epoxy resin at room temperature (25°C). Using 180–320-grit silicon carbide (SiC) papers, the instruments were then ground from their tips until the D5 level of the instruments was exposed. Cross-sectional SEM images of the instruments were then captured (Figure 2). The cross-sectional area of each instrument was calculated using AutoCAD software (Autodesk, San Rafael, CA).

### Statistical analysis

Data were first analyzed using the Shapiro–Wilk test to verify the assumption of normality. One-way ANOVA and post hoc Tukey tests were then performed using SPSS 21.0 (IBM-SPSS Inc. Chicago, IL). Statistical significance was set at 5%.

## Results

### Cyclic fatigue resistance test

The means and standard deviations of the NCF values, as well as the lengths of the fractured segments, are shown in Table 1. All the ﬁles fractured ﬁrst in the apical curvature and then in the coronal curvature. In both the apical and coronal curvatures, the NCF of the GF was signiﬁcantly higher than that of the RPC Blue and WOGfiles (P<0.05). In contrast, the cyclic fatigue resistance of the RPC Blue and WOG files in the apical and coronal curvatures was similar (P>0.05). There was no signiﬁcant difference between the GF, WOG files, and Reciproc Blue ﬁles with respect to the lengths of the fractured ﬁle fragments in either the apical or coronal curvature (P>0.05).

**Table 1:** The number of cycles to failure and length (mm) of fractured fragments of instruments during static cyclic fatigue testing in double curvature

		**Apical Curvature**		**Coronal Curvature**	
**Group**	**No.**	**Number of Cycles to Fracture**	**Fracture Length**	**Number of Cycles to Fracture**	**Fracture Length**
**WaveOne Gold**	20	1028.12±164.48^a^	2.37±0.53^a^	1215.65±218.72^a^	6.35±1.22^a^
**Reciproc Blue**	20	987.45±148.05^a^	2.59±0.47^a^	1161.76±174.15^a^	6.48±1.07^a^
**Genius File**	20	4855.22±631.15^b^	2.42±0.49^a^	5730.11±916.85^b^	6.51±1.14^a^
**P-value**		<0.05	>0.05	<0.05	>0.05

* Different superscripts indicate statistically significant difference at 5% significant level.

### Bending resistance test

The means and standard deviations of bending resistance and the cross-sectional areas of the instruments are shown in Table 2. The bending resistance of the GF was significantly higher than that of the RPC Blue and WOG files (P<0.05). There was no difference in the bending resistance between RPC Blue and WOG files (P>0.05).

**Table 2:** The mean and standard deviation of maximum bending resistance (gf) and the cross-sectional area (μm^2^) values of tested instruments.

**Group**	**No.**	**Bending Resistance**	**Cross-Sectional Area**
**WaveOne Gold**	20	349.15±41.88 ^a^	148186±7409 ^a^
**Reciproc Blue**	20	372.07±48.36 ^a^	202549±8101 ^b^
**Genius File**	20	504.78±75.60 ^b^	101479±6088 ^c^
**P-value**		<0.05	<0.05

* Different superscripts indicate statistically significant difference at 5% significant level.

### Determination of the cross-sectional areas of the instruments

There was a significant difference in the cross-sectional areas of instruments. The RPC Blue had the largest cross-sectional area, and the GF had the smallest cross-sectional area (P<0.05).

## Discussion

During clinical use, fractures of NiTi files are mainly the result of cyclic fatigue.^1^ Although many factors potentially affect the cyclic fatigue resistance of files, anatomic variations, such as double curves in the root canal, are of particular importance, and these present a challenge during root canal preparation.^11^ There are no reports in the literature on the cyclic fatigue resistance of GF and RPC Blue files in double-curved canals. Thus, the present study compared the cyclic fatigue resistance and bending resistance of RPC Blue, WOG and GF NiTi systems. According to the results of the present study, the bending resistance and cyclic fatigue resistance of the GF was higher than that of the RPC Blue and WOG files. Thus, both the null hypotheses of the present study were rejected.

The most important disadvantage of laboratory studies examining the cyclic fatigue resistance of NiTi rotary files is that the factors that can affect the study results (cross-sections, sizes and metallurgical properties of files) cannot be completely standardized.^12^ Although examining the cyclic fatigue resistance on extracted teeth is the best method for mimicking the clinical conditions, the teeth cannot be anatomically standardized.^13^ It is almost impossible to find the teeth having appropriate length, width and curvature diameter and angle, for specially working on S-shaped canals.^14^ For this reason, the standard canals artificially made of stainless steel are preferred for cyclic fatigue resistance tests, rather than the teeth.^15,16^ In cyclic fatigue tests on artificial canals, the limitations such as differences between artificial canals and dentin, and the inability of reflecting torsional forces, which the files are exposed to during root canal preparation, on the artificial canals should be kept in mind. For this reason, the results of cyclic fatigue tests on artificial canals should be carefully extended to the clinical conditions.^17^ The files used in the present study firstly fractured in the apical curvature and then in the coronal curvature. This finding is consistent with the previous studies examining cyclic fatigue resistance of NiTi files in S-shaped artificial canals.^18,19^ Moreover, in previous studies, it was shown that cyclic fatigue resistance of NiTi files is influenced by canal radii and angle of curvature.^20,21^ It is thought that the reason for this finding is that the apical curvature radius (2 mm) of the artificial canal used in the present study is smaller than the radius of the coronal curvature (5 mm). In this study, there was no significant difference in the mean lengths of the fractured segments in any of the groups in either the apical or coronal curvature. The fractured length of each ﬁle was at the center of curvature or just below this point, which conﬁrmed the precise trajectory and position of the instruments. The SEM images of the fractured surfaces of the tested files confirmed that the tested files fractured due to cyclic fatigue.

There are no studies on the bending resistance of the GF, RPC Blue and WOG systems. Thus, the results of the present study cannot be directly compared with those of other studies. The cross-sectional area and diameter of the core of NiTi files have a substantial impact on the bending resistance of the files.^22^ In the present study, the cross-section of each instrument was imaged at D5 under SEM, and the area was measured using AutoCAD software. RPC Blue had the largest area (202549±8101 μm^2^), followed by WOG (148186±7409 μm^2^) and GF (101479±6088 μm^2^). The RPC Blue, WOG, and GF systems are manufactured using a thermomechanical process, a gold alloy, and thermally treated NiTi alloy, respectively. Although the GF had the smallest cross-sectional area among the tested files, it also had the highest bending resistance. This finding could be due to differences in the mechanical properties of the type of NiTi alloy in the tested file. Elsaka et al^23^ reported that the bending resistance of WOG was lower than that of RPC Blue. De-Deus et al^24^ found that the bending resistance of RPC Blue was lower than that of RPC. They attributed the lower bending resistance of RPC Blue and WOG to the superior properties of the NiTi alloys from which they are made. According to the results of the present study, there was no significant difference between the bending resistance of RPC Blue and WOG. The similar manufacturing process (thermal treatment) used in the production of these files might explain this finding.

As no previous studies have compared the cyclic fatigue resistance of the GF, RPC Blue and WOG systems, the results of the present study cannot be directly compared with other studies. According to the results of the present study, the GF was more resistant to cyclic fatigue than the RPC Blue and WOG files. However, there was no significant difference between the cyclic fatigue resistance of RPC Blue and WOG files. Özyürek^15^ and Topçuoğlu et al^16^ reported that the cyclic fatigue resistance of WOG was higher than that of RPC and WO. Adıgüzel and Çapar^25^ reported that the cyclic fatigue resistance of WOG was higher than that of WO. The taper of the files might influence the cyclic fatigue resistance of NiTi files. Previous studies demonstrated that the cyclic fatigue resistance of NiTi files increased with decreasing file diameters.^26,27^ Research also showed that decreasing the diameter and metal mass of the instrument at the maximum stressed point improved the cyclic life of NiTi files.^28^ In line with this finding, among the files tested in the present study, the GF showed the highest cyclic fatigue resistance and smallest cross-sectional area at the D5 level. Previous studies showed that the cyclic fatigue life of NiTi files increased with a decrease in the reciprocation angle.^29,30^ In the present study, the reciprocation angle (90° clockwise and 30° counter clockwise) of the GF was lower than that of WOG (150° counter clockwise and 30° clockwise) and RPC Blue (120° counter clockwise and 30° clockwise). The reciprocation angle might also explain the higher cyclic fatigue resistance of the GF system, as compared to the RPC Blue and WOG systems.

## Conclusion

Within the limitations of the present study, the GF NiTi system exhibited the highest cyclic and bending resistance among the experimental groups.

## References

[1] Cheung GS (2007). Instrument fracture: mechanisms, removal of fragments, and clinical outcomes. Endod Top.

[2] Sattapan B, Nervo GJ, Palamara JE, Messer HH (2000). Defects in rotary nickel-titanium files after clinical use. J Endod.

[3] Yang Q, Shen Y, Huang D, Zhou X, Gao Y, Haapasalo M (2017). Evaluation of Two Trephine Techniques for Removal of Fractured Rotary Nickel-titanium Instruments from Root Canals. J Endod.

[4] Peters O, Gluskin A, Weiss R, Han J (2012). An in vitro assessment of the physical properties of novel Hyflex nickel–titanium rotary instruments. Int Endod J.

[5] Ferreira F, Adeodato C, Barbosa I, Aboud L, Scelza P, Zaccaro Scelza M (2016). Movement kinematics and cyclic fatigue of NiTi rotary instruments: a systematic review. Int Endod J.

[6] Brochure RB. http://www.vdw-dental.com/fileadmin/redaktion/downloads/produkte/en/reciprocblue_brochure_EN_rev0.pdf (Access in March 2017).

[7] Brochure WG. https://www.dentsply.com/content/dam/dentsply/pim/manufacturer/Endodontics/Obturation/Gutta_Percha_Points/WaveOne_Gold_Gutta_Percha_Points/W1G_Brochure_EN.pdf (Access in March 2017).

[8] Brochure GF. https://www.ultradent.com/SiteCollectionImages/Multi-Media-Tab/Brochures/Endodontics/Documents/Genius-Files-Brochure.pdf (Access in March 2017).

[9] Topçuoğlu HS, Topçuoğlu G, Akti A, Düzgün S (2016). In Vitro Comparison of Cyclic Fatigue Resistance of ProTaper Next, HyFlex CM, OneShape, and ProTaper Universal Instruments in a Canal with a Double Curvature. J Endod.

[10] Lopes HP, Elias CN, Vieira MV, Siqueira JF, Mangelli M, Lopes WS (2013). Fatigue life of Reciproc and Mtwo instruments subjected to static and dynamic tests. J Endod.

[11] Willershausen B, Kasaj A, Röhrig B, Marroquin BB (2008). Radiographic investigation of frequency and location of root canal curvatures in human mandibular anterior incisors in vitro. J Endod.

[12] Cheung G, Zhang E, Zheng Y (2011). A numerical method for predicting the bending fatigue life of NiTi and stainless steel root canal instruments. Int Endod J.

[13] Yao JH, Schwartz SA, Beeson TJ (2006). Cyclic fatigue of three types of rotary nickel-titanium files in a dynamic model. J Endod.

[14] Saleh AM, Gilani PV, Tavanafar S, Schäfer E (2015). Shaping ability of 4 different single-file systems in simulated S-shaped canals. J Endod.

[15] Özyürek T (2016). Cyclic Fatigue Resistance of Reciproc, WaveOne, and WaveOne Gold Nickel-Titanium Instruments. J Endod.

[16] Topçuoğlu H, Düzgün S, Aktı A, Topçuoğlu G. Laboratory comparison of cyclic fatigue resistance of WaveOne Gold, Reciproc and WaveOne files in canals with a double curvature. Int Endod J 2016 (in press). 10.1111/iej.12674 27344032

[17] Burroughs JR, Bergeron BE, Roberts MD, Hagan JL, Himel VT (2012). Shaping ability of three nickel-titanium endodontic file systems in simulated S-shaped root canals. J Endod.

[18] Al-Sudani D, Grande NM, Plotino G, Pompa G, Di Carlo S, Testarelli L (2012). Cyclic fatigue of nickel-titanium rotary instruments in a double (S-shaped) simulated curvature. J Endod.

[19] Duke F, Shen Y, Zhou H, Ruse ND, Wang Z-j, Hieawy A (2015). Cyclic fatigue of ProFile Vortex and Vortex Blue nickel-titanium files in single and double curvatures. J Endod.

[20] Lopes HP, Gambarra-Soares T, Elias CN, Siqueira JF, Inojosa IF, Lopes WS (2013). Comparison of the mechanical properties of rotary instruments made of conventional nickel-titanium wire, M-wire, or nickel-titanium alloy in R-phase. J Endod.

[21] Inan U, Aydin C, Tunca YM (2007). Cyclic fatigue of ProTaper rotary nickel-titanium instruments in artificial canals with 2 different radii of curvature. Oral Surg Oral Med Oral Pathol Oral Radiol Endod.

[22] Zhang E-W, Cheung GS, Zheng Y-F (2010). Influence of cross-sectional design and dimension on mechanical behavior of nickel-titanium instruments under torsion and bending: a numerical analysis. J Endod.

[23] Elsaka S, Elnaghy A, Badr A. Torsional and bending resistance of WaveOne Gold, Reciproc and Twisted File Adaptive instruments. Int Endod J 2016 (in press). 10.1111/iej.12728 27917513

[24] De-Deus G, Silva EJNL, Vieira VTL, Belladonna FG, Elias CN, Plotino G (2017). Blue Thermomechanical Treatment Optimizes Fatigue Resistance and Flexibility of the Reciproc Files. J Endod.

[25] Adıgüzel M, Capar ID. Comparison of Cyclic Fatigue Resistance of WaveOne and WaveOne Gold Small, Primary, and Large Instruments. J Endod 2017 (in press). 10.1016/j.joen.2016.11.021 28216272

[26] Capar ID, Kaval ME, Ertas H, Sen BH (2015). Comparison of the cyclic fatigue resistance of 5 different rotary pathfinding instruments made of conventional nickel-titanium wire, M-wire, and controlled memory wire. J Endod.

[27] Parashos P, Gordon I, Messer HH (2004). Factors influencing defects of rotary nickel-titanium endodontic instruments after clinical use. J Endod.

[28] Grande N, Plotino G, Pecci R, Bedini R, Malagnino V, Somma F (2006). Cyclic fatigue resistance and three‐dimensional analysis of instruments from two nickel–titanium rotary systems. Int Endod J.

[29] Gambarini G, Rubini AG, Al Sudani D, Gergi R, Culla A, De Angelis F (2012). Influence of different angles of reciprocation on the cyclic fatigue of nickel-titanium endodontic instruments. J Endod.

[30] Saber SEDM, El Sadat SMA (2013). Effect of altering the reciprocation range on the fatigue life and the shaping ability of WaveOne nickel-titanium instruments. J Endod.

